# Butyrate and bioactive proteolytic form of Wnt-5a regulate colonic epithelial proliferation and spatial development

**DOI:** 10.1038/srep32094

**Published:** 2016-08-26

**Authors:** Kazuhiko Uchiyama, Toshio Sakiyama, Takumu Hasebe, Mark W. Musch, Hiroyuki Miyoshi, Yasushi Nakagawa, Tong-Chuan He, Lev Lichtenstein, Yuji Naito, Yoshito Itoh, Toshikazu Yoshikawa, Bana Jabri, Thaddeus Stappenbeck, Eugene B. Chang

**Affiliations:** 1 Department of Medicine, University of Chicago, Chicago, IL 60637; USA; 2 Department of Molecular Gastroenterology and Hepatology, Kyoto Prefectural University of Medicine, Kyoto 6028566; Japan; 3 Kagoshima University Graduate School of Medical and Dental Sciences, Kagoshima 8908520; Japan; 4 Department of Pathology, Washington University at St. Louis, St. Louis, MO, USA; 5 Department of Surgery, University of Chicago; Chicago, IL 60637; USA; 6 Department of Gastroenterology, Soroka University Medical Center, Beer-Sheva 84101; Israel

## Abstract

Proliferation and spatial development of colonic epithelial cells are highly regulated along the crypt vertical axis, which, when perturbed, can result in aberrant growth and carcinogenesis. In this study, two key factors were identified that have important and counterbalancing roles regulating these processes: pericrypt myofibroblast-derived Wnt-5a and the microbial metabolite butyrate. Cultured YAMC cell proliferation and heat shock protein induction were analzyed after butryate, conditioned medium with Wnt5a activity, and FrzB containing conditioned medium. *In vivo* studies to modulate Hsp25 employed intra-colonic wall Hsp25 encoding lentivirus. To silence Wnt-5a *in vivo,* intra-colonic wall Wnt-5a silencing RNA was used. Wnt-5a, secreted by stromal myofibroblasts of the lower crypt, promotes proliferation through canonical β-catenin activation. Essential to this are two key requirements: (1) proteolytic conversion of the highly insoluble ~40 kD Wnt-5a protein to a soluble 36 mer amino acid peptide that activates epithelial β-catenin and cellular proliferation, and (2) the simultaneous inhibition of butyrate-induced Hsp25 by Wnt-5a which is necessary to arrest the proliferative process in the upper colonic crypt. The interplay and spatial gradients of these factors insures that crypt epithelial cell proliferation and development proceed in an orderly fashion, but with sufficient plasticity to adapt to physiological perturbations including inflammation.

Cell proliferation and differentiation are highly regulated and ordered processes in the intestinal tract which depend on a complex array of signaling molecules that provide the gut with the ability to respond to a wide range of physiological and pathophysiological perturbations. When activated inappropriately, the same signals can cause or contribute to malignant transformation and the development of colorectal cancers. Wnts, for instance, which comprise a large family of evolutionarily conserved cysteine-rich stem cell growth factors, are differentially expressed throughout the gut and provide the necessary cues for normal epithelial cell development^1^,^2^. Several major Wnts are expressed and secreted by stromal pericrypt myofibroblasts that form in a syncitial sheath that contact the extracellular matrix underlying colonic epithelial cells of the lower third to one half of colonic crypts^3^. These cells are believed to be important to the stem and proliferative cell compartments, but would have a negative impact on the maturation of cells in the upper third of the crypt and surface epithelium. In fact, the containment of these cues to the lower crypt is inherently important for allowing the maturational process of the upper crypt to proceed. The restricted expression of Wnts to the lower crypt could be in part due to their highly insoluble nature which limits diffusion and actions to the immediate environment. On the other hand, this same property raises questions about how Wnts can function as paracrine factors in light of their inefficient secretion, relative insolubility, and propensity to adhere to the extracellular matrix^1^,^2^,^4^.

Enteric microbes also play a role in regulating host functions and gene expression of the gut epithelium^5^. In germ-free mice, for instance, angiogenesis and mucosal maturation appear to be partially arrested, but can be restored by recolonization by both undefined and defined microbiota^6^. Although there is a limited understanding of mediators of host-microbial interactions, short chain fatty acids (SCFA), resulting from colonic microbial metabolism of dietary fiber or unabsorbed carbohydrates, are prime examples of bioactive molecules that can have profound effects on host responses. Butyrate, for instance, is produced in prodigious amounts and is one of the major anions found in the luminal fluid^7^. Butyrate has many trophic actions on gut mucosa, including regulation of mucosal mass, anti-carcinogenic effects, and maturational properties^8^. Butyrate also induces and maintains the physiological expression of intestinal epithelial heat shock proteins (Hsp) such as Hsp25 (Hsp27 as the human homolog). This induction is a prime example of host-microbe interaction, because Hsp25/27 confers cellular protection, maintains barrier function, inhibits proliferation, and promotes differentiation of colonic epithelial cells^9^,^10^,^11^. Similar effects have been observed in other cell types as well^12^,^13^,^14^,^15^. The expression of colonic epithelial Hsp25/27, however, is region-specific. It is greatest in surface epithelial cells and the cells of the upper one third or half of colonic crypts^10^. In fact, its expression is complementary to the regional distribution of pericrypt myofibroblasts that are found in the lower half of the colonic crypt^3^,^16^,^17^, raising the possibility that these events may be related.

In this study, we report the interaction between pericrypt-derived myofibroblast-derived Wnt-5a and Hsp25/27 in the regulation of colonic epithelial proliferation. We find that pericrypt myofibroblast-derived Wnt-5a is proteolytically converted to a soluble bioactive, 36 mer peptide that likely facilitates its delivery to epithelial Frizzled receptors in the lower part of the colonic crypt to promote cell proliferation and development, an effect mediated through the canonical activation of β-catenin. However this action alone is insufficient and requires simultaneous inhibition of butyrate-induced Hsp25/27 in cells in the proliferative compartment of colonic crypts. As Wnt-5a signaling declines in the upper crypt that lies outside of the pericrypt myofibroblast sheath, butyrate-induced Hsp25/27 expression becomes more prominent as a result of diminishing negative regulation by Wnt-5a.

## Results

### Pericrypt myofibroblasts restore intestinal epithelial proliferation inhibited by butyrate

**Figure 1:**
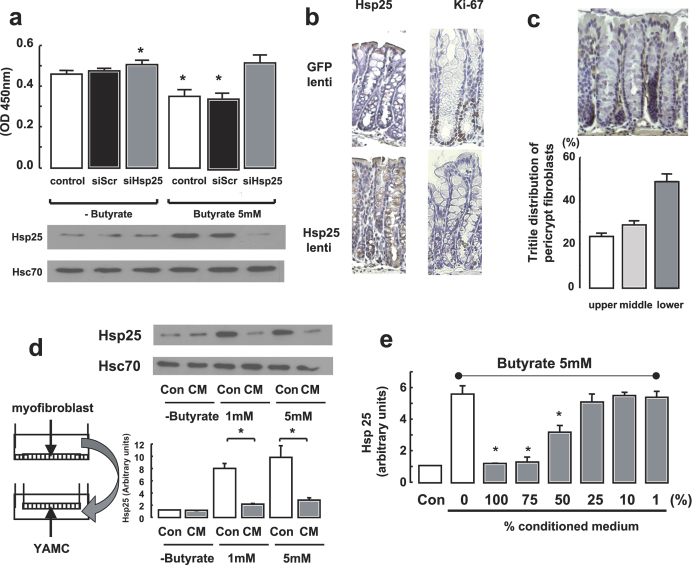
Butyrate inhibition of colonic epithelial cell growth is dependent on induction of Hsp25. (**a**) Butyrate inhibition of cell growth (clear bar, fourth from left) is blocked by silencing Hsp25 (grey bar, far right), but not by scrambled (non-sense) siRNA (siScr). Cell proliferation was measured by the WST-1 assay. **(b)** Lentiviral-induced expression of Hsp25 (Hsp25 lenti) in mouse colon decreases expression of the proliferation marker Ki-67 (brown staining). As a control, empty cassette GFP lentivector (GFP-lenti) was administered the same way. After one hour exposure of the colonic mucosa to luminally-administered lentivirus, the colonic mucosal segment was marked, returned to the abdominal cavity, and then harvested 3 days later, as described in methods. **(c)** *Upper panel*: Alpha smooth muscle actin staining was used to identify peri-crypt myofibroblasts (brown immunostaining). *Lower panel:* Distribution of pericrypt myofibroblast, identified by alpha smooth muscle actin immunostaining, among the upper, middle, and lower tritiles (thirds) of colonic crypts. **(d)** Intestinal myofibroblast-derived conditioned medium (CM) blocks butyrate-stimulated (1 and 5 mM) Hsp25 expression in intestinal epithelial YAMC monolayers. No effects were seen on Hsc70 protein expression which is used as a loading control (d, upper panel). *p < 0.01, n = 5. Con, control cells not treated with conditioned media. *p < 0.01, n = 5. **(e)** Intestinal myofibroblast-derived conditioned medium blocks butyrate-induced (5 mM) Hsp25 in YAMC cells in a concentration-dependent manner.

The short chain fatty acid (SCFA), butyrate (5 mM), inhibits proliferation of murine colonic epithelial YAMC cells measured by the WST-1 assay (Fig. 1a). These diploid, non-transformed cells were grown under permissive conditions favoring their proliferation^18^. The butyrate effect was associated with the induction of Hsp25, (Fig. 1a). The effects of butyrate and Hsp25 on intestinal epithelial proliferation are related, as siRNA silencing of butyrate-stimulated Hsp25 negates the anti-proliferative actions of this SCFA. No effects were seen using a scrambled siRNA. As a corollary experiment, Hsp25 expression was induced in YAMC intestinal epithelial cells by lentivirus, inhibiting cell growth (Fig. S1a). The *in vivo* effects of Hsp25 expression on crypt cell proliferation can be seen in a segment of proximal colonic mucosa exposed to luminally-administered Hsp25 transgene-containing lentivirus. Normally, Hsp25 expression is confined to the upper third of colonic crypts^10^. However, after Hsp25 lentivirus delivery, robust Hsp25 protein expression was observed throughout the colonic crypt epithelium, associated with decreased Ki-67 antibody staining, a marker of cellular proliferation (Fig. 1b). On the other hand, colonic Hsp25 expression and Ki-67 staining were normal in mice that underwent the same procedure, but using a GFP-only lentivirus (Fig. 1b). In the colonic mucosa of global Wnt5a^−/−^ mice, robust Hsp25 protein expression was observed by immunostaining throughout the colonic crypt epithelium (Fig. S2a), associated with decreased Ki-67 antibody staining (Fig. S2b). On the other hand, colonic Hsp25 expression and Ki-67 staining were normal in control mice. Colonic epithelial expression of Hsp25 *in vivo* is highly dependent on microbial cues, particularly the production of short chain fatty acids (SCFA)^7^. Given the sensitivity of Hsp25 gene expression to even low levels of SCFAs^10^, Hsp25 protein expression should be detectable throughout the entire colonic crypt. As this is not the case, we considered the possibility that Hsp25 gene expression in the lower part of the colonic crypt is under negative regulation, possibly by stromal pericrypt myofibroblasts, which have a pattern of distribution inversely related to Hsp25 expression and are found predominantly in the lower tritile (third) of the crypt (Fig. 1c). To test this possibility, conditioned media (CM) from murine VUPF intestinal myofibroblast cells were applied to the basal side of YAMC monolayers on Transwell supports (Fig. 1d). VUPF cells are alpha-smooth muscle actin positive myofibroblasts from the mouse colon. Butyrate, at both 1 and 5 mM, induced Hsp25 expression in YAMC cells (Fig. 1d, lanes 3 and 5). This effect, however, was inhibited by myofibroblast-derived CM in a concentration-dependent manner (Fig. 1d,e). The mechanism underlying the effect appears to be through inhibition of butyrate-induced mRNA expression of Hsp25 (Fig. S3a). These findings are consistent with the secretion of a soluble factor by VUPF myofibroblasts that negatively regulates butyrate-induced Hsp25 expression in intestinal epithelial cells.

### Wnt-5a mediates the effects of pericrypt myofibroblast on colonic epithelial proliferation and inhibition of butyrate-stimulated Hsp25

**Figure 2:**
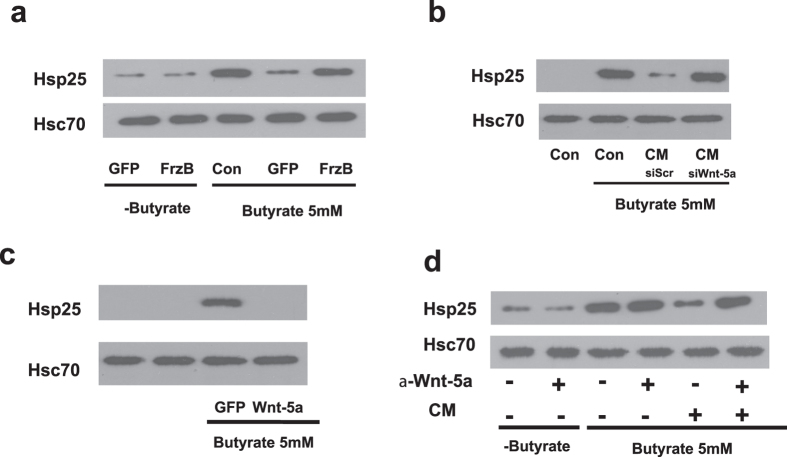
Myofibroblast-derived Wnt-5a inhibits butyrate effects on colonic epithelial YAMC Hsp25. (**a)** Representative Western blot showing that the overexpression of the Wnt-binding protein FrzB in pericrypt VUPF myofibroblasts is associated with decreased inhibitory ability of conditioned media on butyrate-induced epithelial YAMC Hsp25 protein expression (far right lane). GFP – Conditioned media from myofibroblast cells treated with green fluorescent protein label-empty adenovirus vector. FrzB – myofibroblast cells treated with GFP-FzB adenoviral vector. Con – control cells that were not treated with viral vectors. **(b)** RNA-silencing of Wnt-5a (siWnt-5a) in VUPF myofibroblasts significantly reduces the inhibitory effects of conditioned media (CM) on butyrate-stimulated Hsp25 expression in YAMC cells (Con). Treatment with scrambled siRNA (siScr) had no effects. **(c)** Conditioned medium from Wnt-5a transfected HEK-293 cells inhibits butyrate–induced Hsp25 protein expression in YAMC cells. GFP – conditioned media from HEK293 cells that were treated with empty GFP adenoviral vector. **(d)** Treatment of VUPF conditioned medium (CM) with an antiserum to Wnt-5a (α-Wnt-5a) blocks its ability to inhibit butyrate induction of Hsp25.

Wnts are among many myofibroblast-derived factors that regulate epithelial cell proliferation and development^2^,^19^. To examine their role in mediating myofibroblast inhibition of butyrate-induced Hsp25, FrzB or GFP (as a negative control and to monitor viral infection rates) were expressed in VUPF myofibroblasts using an adenovirus vector. FrzB is a Wnt-binding protein that competitively inhibits Wnt binding to the Frizzled receptor^20^. CM from FrzB-expressing myofibroblasts had no effects alone on basal Hsp25 expression in absence of butyrate (Fig. 2a, second lane), but negated the inhibitory actions of VUPF CM on butyrate-stimulated Hsp25 expression (Fig. 2a, far right lane). In contrast, CM from myofibroblasts infected only with empty cassette Ad-GFP significantly inhibited butyrate-stimulated Hsp25 induction. These findings therefore implicated Wnts in mediating the actions of myofibroblast CM on butyrate-induced intestinal epithelial Hsp25 expression and inhibition of proliferation.

The profile of Wnt gene expression was next assessed by PCR (Fig. S3b). Among the several Wnts detected, Wnt-5a mRNA was readily detectable, consistent with previous findings using *in situ* hybridization^2^. As further confirmation, Wnt-5a immunoreactivity was also primarily observed in pericrypt myofibroblasts of the lower half of normal murine colonic crypt (Fig. S4). To test the role of Wnt-5a in mediating the effects of VUPF CM, experimental perturbations of Wnt-5a expression in VUPF myofibroblasts were performed. The inhibitory effects of VUPF-CM on butyrate-stimulated Hsp25 were blocked by treating the VUPF cells with siRNA to Wnt-5a, but not a scrambled control siRNA (Fig. 2b). Conditioned media were then prepared from human epithelial kidney HEK293 cells (which lack basal Wnt-5a expression) that had been infected with a Wnt-5a - or GFP-adenoviral vector. Only conditioned media from Wnt-5a-expressing HEK293 cells inhibited butyrate-stimulated YAMC Hsp25 expression (Fig. 2c). The inhibitory effects of VUPF-CM on butyrate-stimulated Hsp25 expression were also inhibited in the presence of anti-Wnt-5a blocking antibody (AF645; R&D Systems) (Fig. 2d). Collectively, these data implicated Wnt-5a as the primary negative regulator of butyrate-induced Hsp25 expression secreted by pericrypt myofibroblasts.

**Figure 3:**
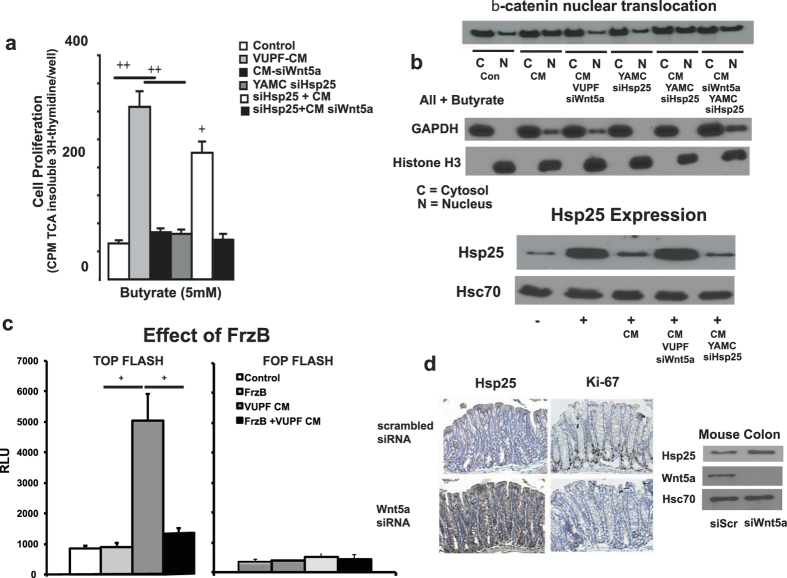
Wnt-5a mediates the actions of myofibroblast on butyrate-inhibited intestinal epithelial cell growth through activation of the canonical β-catenin pathway. (**a**) RNA silencing of VUPF myofibroblast Wnt-5a expression (siWnt-5a) blocks the proliferative effects of VUPF conditioned media (CM). Proliferation was measured by rates of ^3^H-thymidine incorporation. ^++^p < 0.01, n = 5 (**b**) Western blot showing that myofibroblast-derived conditioned medium increases nuclear localization of β-catenin in butyrate-stimulated YAMC cells, an effect that is inhibited by pre-treating VUPF cells with siRNA to Wnt-5a (siWnt-5a). All conditions were under butyrate treatment (5 mM); C- cytosol fraction; N – nuclear fraction. (**c**) VUPF conditioned media-induced activation of β-catenin, assessed by TOPflash reporter, is blocked by FrzB. The negative control FOP Flash is presented for all four conditions. (**d**) *In vivo* silencing of colonic mucosal Wnt-5a (siWnt-5a) is associated with pronounced epithelial Hsp25 expression (brown staining, lower left panel) and decreased Ki67 staining (lower right panel) of colonic crypts. In contrast, intramural injection of scrambled siRNA (siSrc) did not affect typical pattern of Hsp25 or Ki67 immunostaining (upper left and right panels, respectively). Shown below, *in vivo* expression of murine colonic mucosal Wnt-5a is inhibited by intramural injection of siRNA to Wnt-5a. Injection with scrambled siRNA (siScr) was used as a control. Silencing of colonic mucosal Wnt-5a is associated with increased protein expression of Hsp25, but no change in Hsc70.

We next addressed whether Wnt-5a of VUPF-CM modifies butyrate-inhibited cell proliferation and induces activation and nuclear translocation of β-catenin in YAMC cells. Wnt-5a may activate the canonical pathway, stimulating β-catenin translocation to the nucleus as well as non-canonical planar polarity and the Ca^++^ dependent pathways. CM from VUPF myofibroblasts treated with Wnt-5a siRNA failed to block the anti-proliferative actions of butyrate measured by ^3^H-thymidine incorporation (Fig. 3a). We next examined the effects of VUPF CM on nuclear localization of β-catenin in butyrate (5 mM)-treated YAMC cells. VUPF-CM stimulated nuclear translocation of β-catenin which was associated with decreased butyrate-stimulated Hsp25 protein expression in YAMC cells (Fig. 3b). In contrast, conditioned media from VUPF cells where Wnt-5a was silenced failed to stimulate β-catenin nuclear translocation in the presence of butyrate (Fig. 3b). This was associated with a return in butyrate-stimulated Hsp25 expression (Fig. 3b). CM had no effect when YAMC cell Hsp25 was silenced (Fig. 3b). Similarly, an independent measure of β-catenin activation using a TOPflash β-catenin reporter (with tandem TCF binding elements), VUPF-CM significantly activates β-catenin, but this effect is blocked when VUPF are transfected to express FrzB (Fig. 3c). No stimulation with VUPF CM was observed with the negative control FOP Flash luciferase reporter (Fig. 3C). Collectively, these data indicated that pericrypt myofibroblast Wnt-5a stimulates proliferation of butyrate-treated YAMC cells through inhibition of induced Hsp25 expression and canonical activation of β-catenin.

We next examined the *in vivo* role of Wnt-5a as a negative regulator of intestinal epithelial Hsp25 expression and in promoting crypt cell proliferation. Wnt-5a or scrambled siRNA was injected into the colonic wall of anesthetized mice, using a previously described approach^21^. This region was then harvested 72 hrs later and processed for immunohistochemistry and Western blot analysis. Wnt-5a siRNA, but not scrambled siRNA (siScr), effectively inhibited Wnt-5a protein expression in mucosal scrapings (Fig. 3d, immunoblot), which was associated with increased expression of Hsp25. By immunohistochemistry, increased Hsp25 expression was observed throughout epithelial cells of the intestinal crypt that was associated with a significant decrease in Ki67 immunostaining, the latter a marker of crypt cell proliferation (Fig. 3d). Scrambled siRNA did not affect the normal expression of Hsp25 or Ki-67 protein expression.

### Proteolytic processing of Wnt-5a to a soluble, bioactive, 36 mer peptide that promotes intestinal epithelial proliferation through β-catenin activation and inhibits butyrate-stimulated Hsp25

**Figure 4:**
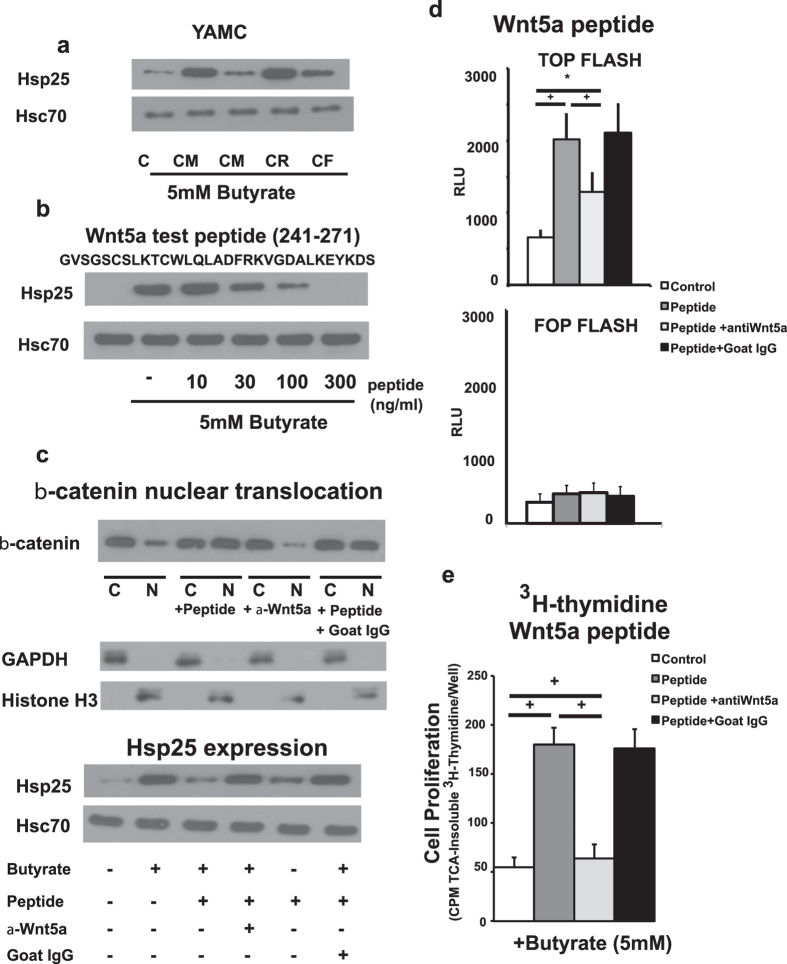
Bioactivity resides in cleaved peptide fragment of Wnt-5a. **(a)** The bioactivity of conditioned media for inhibition of butyrate-induced Hsp25 expression in YAMC cells (CM) resides in the <10 kDa Centricon filtrate (CR), but not in the >10 kDa retentate (CR). Con – control YAMC cells that were not treated with conditioned media. **(b)** Based on tandem mass spectrometry analysis of the <10 kDa fraction, the near full length Wnt-5a peptide sequence (aa241 to aa271) was synthesized (the underlined portion of this sequence indicates part of the epitope to which the Wnt-5a antibody was made). Shown by Western blot, the synthesized Wnt-5a peptide inhibited butyrate-stimulated Hsp25 protein expression in YAMC cells in a dose-related fashion. Hsc70 is shown as a loading control. **(c)** Western blots showing that the antibody to Wnt-5a (α-Wnt-5a) blocks the action of the Wnt-5a synthesized peptide on β-catenin nuclear translocation (upper panel) and butyrate-stimulated Hsp25 expression in YAMC cells (lower panel). Goat IgG was used as the non-immune serum control. Fractions were analyzed for GAPDH and histone H3 to assess purity of nuclear and cytoplasmic separation. **(d)** The Wnt-5a synthesized peptide activates β-catenin, demonstrated by TOPflash reporter activity, This activity is blocked by Wnt-5a antibody, but not by goat IgG. The negative control FOP Flash is presented for all four conditions. **(e)** The Wnt-5a synthesized peptide blocks butyrate-inhibited cellular proliferation of YAMC cells (measured by the ^3^H-thymidine incorporation). The actions of Wnt-5a synthesized peptide are blocked by anti-Wnt-5a antibody. ^+^p < 0.01, n = 5.

The Wnt-5a gene encodes a protein of approximately 40 kDa molecular weight^20^. However, when the VUPF-CM was subjected to Centricon filtration with a 10 kDa cutoff, the bioactivity (inhibition of butyrate-induced YAMC Hsp25) was primarily found in the filtrate (CF) and not in the retentate (CR) (Fig. 4a). Because this finding was unanticipated, the bioactive fraction was subjected to purification, and analyzed by tandem mass spectrometry (Chicago Biomedical Consortium Proteomics and Informatics Services Facility). A peptide with a neutral mass of 3903.3434 was obtained from MS/MS spectra. This information was processed using the Molecular Feature Extractor and subjected to a mass based search against the sequence of mouse Wnt-5a (accession NM_009524) using a random cleavage fit using the program, GPMAW7.1, with an error tolerance of 5 ppm. The results matched to the sequence of aa241 to aa276 of Wnt-5a - GVSGSCSLKTCWLQLADFRKVGDALKEKYDSAAAMR (the underlined portion of this sequence indicates part of the epitope to which the Wnt-5a antibody was made). This region of Wnt-5a is highly conserved among Wnts and its formation could be a result of enzymatic cleavage by serum or cellular tryptase (cutting at the N-terminal basic N-terminal histidine and C-terminal arginine).

To test the functional properties of this peptide, the Wnt-5a peptide region from aa241 to aa271 (GVSGSCSLKTCWLQLADFRKVGDALKEKYDS) was synthesized and purified by HPLC (purity 92.7%). This shorter peptide was made because of the difficulty of preparing and purifying the full length 36 mer endogenous Wnt-5a peptide by the commercial vendor. As shown in Fig. 4b, the Wnt-5a synthesized peptide inhibits butyrate-stimulated Hsp25 expression in YAMC cells in a concentration-dependent fashion. The blocking antibody also inhibited Wnt-5a peptide-induced nuclear translocalization and activation of β-catenin in butyrate (5 mM)-stimulated YAMC cells (Fig. 4c,d). For the activation of β-catenin measured by the TOP Flash luciferase reporter system, no stimulation of the negative control FOP Flash was observed (Fig. 4d). Wnt-5a synthesized peptide’s action on butyrate-stimulated Hsp25 expression in YAMC cells is also inhibited by the presence of anti-Wnt-5a antibody (Fig. 4c, lower panel). Butyrate-inhibited YAMC cell proliferation was also significantly reversed by the Wnt-5a synthesized peptide (Fig. 4e). This action could be negated with anti-Wnt-5a blocking antibody. In contrast to its effects on VUPF-CM, FrzB did not block the actions of Wnt-5a peptide on YAMC proliferation or activation and nuclear localization of β-catenin (Fig. S5a–c). We attributed these negative findings in this case to subtle differences between the endogenous 36 mer and synthesized 31 mer Wnt-5a peptides that may affect FrzB binding.

**Figure 5:**
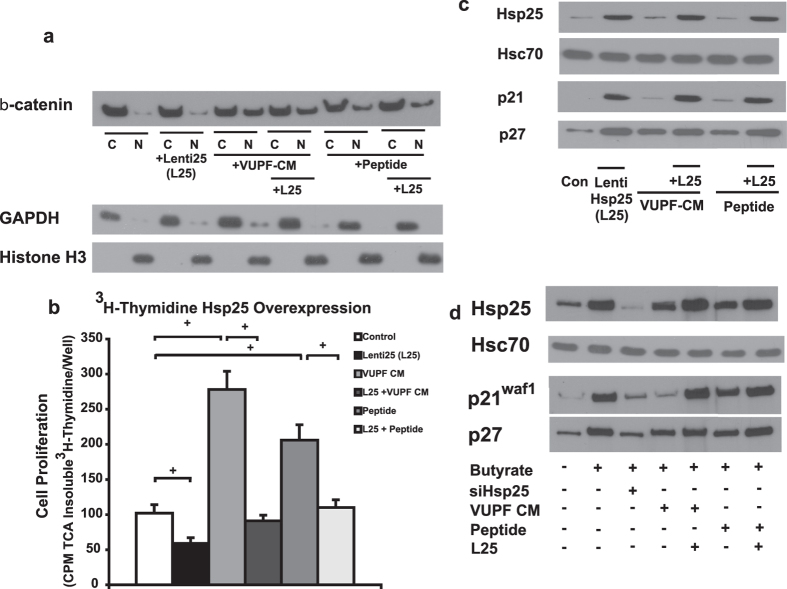
Butyrate-stimulated or constitutive expressed Hsp25 inhibits YAMC cell proliferation, but not through inhibition of Wnt-5a induced β-catenin activation. (**a**) Western blot for β-catenin shown that constitutive expression of Hsp25 achieved through lentiviral delivery (L25) to YAMC cells has no effect on nuclear translocation of β-catenin induced by VUPF conditioned media (VUPF-CM) or synthesized Wnt-5a peptide. No butyrate was present under these conditions. C – cytosol fraction; N – nuclear fraction. Fractions were analyzed for GAPDH and histone H3 to assess purity of nuclear and cytoplasmic separation. (**b**) Constitutive expression of Hsp25 achieved through lentiviral delivery inhibits YAMC cell proliferation stimulated by myofibroblast conditioned media (VUPF-CM) and Wnt-5a peptide. Cell proliferation was determined by 3H-thymidine incorporation. ^+^p < 0.01, n = 5. (**c**) Immunoblots showing butyrate (5 mM) and lentiviral Hsp25-induced expression (L25) both stimulate increases in p21^waf-1^ and p27 protein expression, two cell cycle regulators known to cause G1 arrest. VUPF myofibroblast conditioned media and Wnt-5a peptide by themselves (in absence of butyrate) do not affect p21^waf-1^ and p27 expression (**c**), but inhibit butyrate stimulated increases in Hsp25, p21^waf-1^ and p27.

We next determined the effects of Hsp25 overexpression in YAMC cells on CM- and Wnt-5a synthesized peptide-induced nuclear localization of Wnt-5a (Fig. 5a). By doing so, we reproduce the butyrate effect, but through a mechanism that should be resistant to inhibition by CM- or Wnt-5a synthesized peptide. Hsp25 expression in YAMC cells was increased through Hsp25 lentiviral infection (see Fig. S1a), which did not affect states of β-catenin nuclear localization under basal or conditions of VUPF-CM or Wnt-5a synthesized peptide stimulation (Fig. 5a). Both VUPF-CM and Wnt-5a synthesized peptide stimulate YAMC proliferation (Fig. 5b), although less well by the latter. Overexpression of Hsp25 with lentiviral delivery was able to override the cell proliferation stimulated by both VUPF-CM and Wnt-5a peptide. These data strongly suggest that Hsp25 exerts its antiproliferative effects at steps distal to Wnt-5a-induced activation and nuclear localization of β-catenin. Based on previous reports^11^,^22^, we examined if this could be through Hsp25 induction of two cell cycle regulatory proteins, p21^waf-1^ and p27. Both proteins bind to and inhibit the activity of cyclin-CDK2 or -CDK4 complexes, and thus function as a regulator of cell cycle progression at G_1_. Hsp25 induction through lentiviral delivery (L25) induces expression of both p21^waf-1^ and p27 under basal conditions (Fig. 5c). Both VUPF-CM and Wnt-5a synthesized peptide had no effects on the basal expression of these proteins, and lentiviral-induced Hsp25 expression is still able to induce p21^waf-1^ and p27 under these conditions. Shown in Fig. 5d, the expression levels of p21^waf-1^ and p27 are increased in butyrate-stimulated YAMC cells compared to control cells in absence of butyrate. When the same cells are treated with VUPF-CM or Wnt-5a synthesized peptide, p21^waf-1^ and p27 expression are inhibited, albeit not as robustly with the Wnt-5a synthesized peptide. In YAMC cells where Hsp25 expression is increased through lentiviral delivery, the expression of p21^waf-1^ and p27 are increased even in the presence of VUPF conditioned media or Wnt-5a synthesized peptide. These data suggest that the induction of Hsp25 by butyrate exerts an anti-proliferative effect by specifically inducing the expression of cell cycle regulatory proteins, i.e. at a step distal to the activation and nuclear localization of β-catenin. Thus, in the lower part of the colonic crypt, the balance between Wnt-5a and butyrate favors the former, promoting cell proliferation. In contrast, in the upper regions of the colonic crypt where there is diminishing Wnt-5a signal due to fewer pericrypt myofibroblasts, the balance favors butyrate, resulting in cessation of epithelial proliferation and promotion of cell maturation.

### Wnt-5a peptide speeds healing of experimental colitis

**Figure 6:**
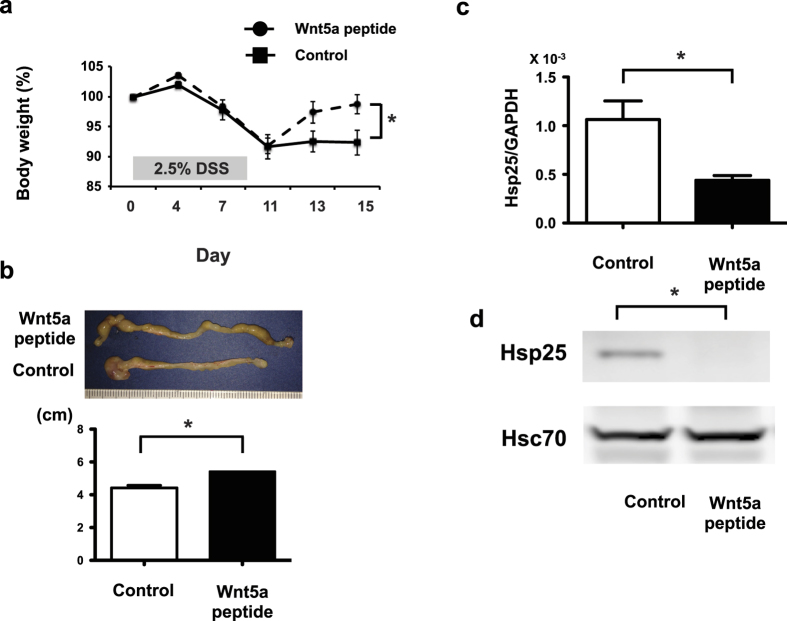
Wnt-5a peptide enhances recovery after DSS-induced colitis and suppresses Hsp25 mRNA. Mice were treated with 2.5% (wt/vol) DSS in drinking water for 7 days and then half treated with 36mer Wnt-5a peptide. Body weights were measured during recovery and after one week of daily peptide injections, all mice were sacrificed and colon length measured, followed by RNA and protein extraction and analysis for Hsp25. Data are means ± SEM for 5 mice in each group. *p < 0.01 compared with non-peptide treated mice by paired Student’s T test.

In light of the actions of Wnt-5a peptide, we examined its biological actions in the context of experimental colitis induced by dextran sulfate sodium (DSS). To determine if the Wnt-5a peptide might prevent the damage or subsequent recovery that involves a wound healing response, mice were treated with DSS and then allowed to recover with or without Wnt-5a peptide. Mice not treated with the peptide did not recover from the loss of body weight 17 days after DSS removal while those treated with the peptide demonstrated full recovery of body weight (Fig. 6). Colon length, which correlates with the degree of colitis, was also significantly longer in mice treated with the peptide after induction of colitis by DSS. Hsp25 mRNA and protein expression were also measured and found decreased in mice treated with Wnt-5a peptide. (Fig. 6c,d).

## Discussion

In this study, we report the unique relationship between two key gut regulatory factors, one host- and the other microbe-derived, which appear to have counterbalancing roles in determining epithelial development from stem cells at the base to the surface epithelium of the colonic crypt. We find that Wnt-5a, secreted as a soluble molecule by pericrypt myofibroblasts, stimulates intestinal epithelial proliferation through a canonical activation of β-catenin (see model presented in Fig. S6a). However, this action by itself is not sufficient and requires the inhibition of butyrate-stimulated Hsp25/27 which, when present, counteracts the Wnt-5a and β-catenin-dependent stimulation of cell proliferation. In contrast, in the upper part of the colonic crypt where Wnt-5a signaling and the presence of pericrypt myofibroblast diminish, the expression of butyrate-stimulated Hsp25/27 is progressively restored as cells migrate to the colonic surface. Under these conditions, any further cell proliferation is inhibited through butyrate stimulated increases in p21^waf-1^ and p27 and the maturational process begins. Thus, the regional confinement of Wnt-5a signaling and interaction with butyrate are important in maintaining the orderly development and turnover of colonic epithelial cells (Fig. S6b). On the same note, perturbations in either of these processes could also have profound effects that disrupt normal development of the gut mucosa. For instance, changes in the enteric microbiota structure or functional metagenome caused by pathogens^23^, change in diet^24^,^25^, or presence of mucosal inflammation^26^ could reduce fermentative capacity and bioavailability of SCFAs. Several studies have shown that this can lead to reduced expression of Hsp25^27^ and other key factors such as intestinal alkaline phosphatase^28^ on which the well being of colonic epithelial cells rely. The relationship between colorectal cancer and low fiber western diets^29^ may also be an example where the countering effects of SCFAs are sufficiently reduced to allow aberrant Wnt-driven proliferation to proceed unchecked. On the host side, increased Wnt signaling as a result of mutational events in stromal or colonic stem cells can readily overwhelm the counterbalancing actions of butyrate-producing bacteria, resulting in conditions favorable for the development of colorectal cancers. Mutations have been found in a number of genes in the Wnt transduction pathway including adenomatous polyposis coli (APC) axin1 and axin2, beta catenin (CTNNB1 gene), and TCF4^30^,^31^. Whether butyrate inhibits growth in the presence of these mutations is unknown.

Predicting how butyrate effects growth and differentiation *in vivo* must consider the conditions ie. both positive and negative growth regulators. Butyrate effects proliferation and differentiation of normal and neoplastic intestinal epithelial cells differently. In colon cancer cells studied *in vitro*, butyrate induces cell cycle arrest, differentiation, and apoptosis, but stimulates proliferation of normal colonocytes^32^,^33^. This has been termed the butyrate paradox. Butyrate is avidly metabolized by intestinal epithelial cells. In neoplastic cells increased anaerobic glycolysis occurs, potentially leading to increased cell butyrate. At these increased concentrations, butyrate has greater effect on histone deacetylase activity that may inhibit their growth^34^,^35^. Our studies used a temperature-sensitive SV40 large T antigen conditionally immortalized mouse colonic cell line, YAMC. Under the conditions tested, butyrate inhibits YAMC cell growth similar to many colon cancer cell lines, but it may not be correct to call these cells cancer or neoplastic as they are SV40 T antigen immortalized. The ability of Wnt-5a to block butyrate induction of Hsp25 could be dependent on state of the cell with respect to proliferation or differentiation. Of note, the *in vitro* cell culture conditions have abundant metabolic substrates and growth factors that may contribute to the effects of butyrate on proliferation and differentiation. *In vivo* the complexity of proliferative and differentiation regulatory factors are likely different from the derivative *in vitro* condition and may be species dependent. Human and mouse embryonic stem cells may demonstrate differences in Wnt/β-catenin signalling and in a context dependent fashion. Therefore in human cells, Wnt proteins other than Wnt-5a may play important roles in growth regulation or Wnt-dependent differentiation.

Another important finding of this study is the discovery that pericrypt myofibroblast-derived Wnt-5a is proteolytically converted to a soluble bioactive peptide that likely facilitates its delivery to epithelial Frizzled receptors in the lower half of the colonic crypt to promote cell proliferation and development, an effect mediated through the canonical activation of β-catenin. Our studies therefore provide a plausible explanation for how pericrypt myofibroblast Wnt-5a and possibly other Wnts function as paracrine growth factors (see Fig. S6a). The process is similar to that reported for *Drosophila* where circular muscle cells of the intestine secrete Wingless, the counterpart to mammalian Wnt, which then crosses the extracellular matrix to control self-renewal of intestinal stem cells (ISCs)^4^. Until this study, it was not apparent how mammalian Wnts of pericrypt fibroblasts could function as paracrine factors in light of their inefficient secretion and high degree of insolubility^36^,^37^,^38^, properties that could be related to lipid modification involving palmitoylation of a conserved cysteine^38^. The conversion of Wnt-5a to a smaller, more soluble bioactive peptide could potentially make its secretion by myofibroblasts more efficient and facilitate its delivery through the extracellular matrix to epithelial Wnt receptors. The Wnt-5a peptide identified by our study (aa241 to aa276) spans one of the most bioactive peptide regions of Wnt-5a previously identified as having the ability to impair migration of human breast epithelial cells^39^. These regions were selected by computer modeling of the most likely exposed amino acid residues – generally representing those that are charged, polar, or accessible to proteolytic cleavage. In essence, the Wnt-5a holoprotein could serve as a type of “toolbox” where specific “tools” (represented by externally located peptide regions) required for a particular cell function are accessed by proteolytic cleavage. The type of Wnt-5a peptide or tool that is selected would then be determined by circumstance, cell type, and by the cellular profile of expressed proteases. Thus, proteins like Wnt-5a could be multi-functional, but specific for a particular occasion. This idea could explain the many different functions and targets attributed to Wnt-5a by various laboratories^23^,^39^,^40^. Wnt-5 may stimulate through the canonical or non-canonical pathway and its action may be cell condition dependent, proliferative versus differentiated.

The process through which Wnt-5a is converted to a smaller bioactive peptide is currently unclear, but may involve intracellular processing or conversion through extracellular tissue or serum tryptases that could emanate from many several potential sources, including epithelial, mesenchymal and mast cells (see proposed model, Fig. S6a,b). Mast cells are a particularly rich source of tryptases which are frequently found near pericrypt fibroblasts^41^,^42^ and are increased in inflammatory diseases such as ulcerative colitis and celiac disease where crypt hyperplasia are commonly observed^43^,^44^.

Finally, we believe the identification of a bioactive, highly soluble Wnt-5a-derived peptide has therapeutic implications for strategies to regulate intestinal stem cells and promote regeneration as after tissue injury^45^. For instance, the synthesis, purification, and delivery of Wnt-5a and related Wnt peptides can be more readily achieved than that for their full length Wnt counterparts. Wnt-derived peptides could also be potentially useful in treating disorders relating to intestinal failure or injury (malnutrition, short bowel syndrome, radiation enteritis, inflammatory bowel diseases). Alternatively, competitive inhibitors of Wnt-5a peptide could be developed to inhibit aberrant stem cell development to prevent or treat disorders arising from intestinal malignancies.

## Materials and Methods

### Cell Culture

Mouse colonic epithelial YAMC cells were maintained as described^18^,^46^. Murine VUPF (Vanderbilt University Pericrypt Myofibroblasts) myofibroblasts were grown at 37 °C in RPMI 1640 with 5% FBS. Both cell lines were a gift of Dr. R. Whitehead (Vanderbilt University, Nashville, TN). The ability of myofibroblasts to modulate butyrate-induced Hsp25 in YAMC cells was determined in three models. First, myofibroblasts were plated on the bottom of permeable supports (confirmed by visual observation) to provide close access to the YAMC cells that were plated on the filter top after fibroblast attachment. Second, VUPF were plated in dishes and YAMC on permeable supports placed on top. In both cases, butyrate was then added to the apical medium. A third model was used where VUPF-conditioned medium was used to treat YAMC monolayers.

### Western blot analysis

Cell proteins were harvested and analyzed by Western blotting as previously described^46^. Antibodies used were anti-Hsp25 antibody (SPA 801; Stressgen, Victoria, BC, Canada), anti-Hsc70 antibody (SPA 815; Stressgen), anti-Wnt-5a antibody (AF645; R&D Systems), anti-actin (rabbit polyclonal, Cell Signalling), anti-smooth muscle actin antibody (A2547; Sigma).

### RNA isolation and reverse transcription

Primers for 19 murine Wnt mRNA were designed using MacVector software (Accelrys, San Diego, CA) or published Wnt primers^47^. Total myofibroblast RNA was extracted using Trizol (Invitrogen) and reverse transcribed using (Superscript II kit, Invitrogen). The two-step quantification cycling protocol was used as previously described^48^.

### Silencing of Wnt-5a and Hsp25 gene expression

YAMC cell Hsp25 was silenced as previously described using a silencing oligonucleotide (bases 539–564, GenBank. NM_013560) designed using RNAi designer (Invitrogen). For VUPF myofibroblast Wnt-5a silencing, a silencing oligonucleotide (exons 4, 5 Ambion ID#187053, Austin, TX) or scrambled oligonucleotide (ID#4611) was used.

### Recombinant Adenoviral Vectors Expressing FrzB, Wnt-5a

For FrzB or Wnt-5a adenovirus, human FrzB and mouse Wnt-5a were PCR-amplified, subcloned into pAdTrack-CMV and recombinant adenovirouses generated^49^. To neutralize Wnt-5a in this medium, rabbit polyclonal anti-Wnt-5a (AF645; R&D Systems) was used. Antibody (1 mg/ml) was added to conditioned medium and gently rotated for 24 hours before use.

### Lentiviral vector production

Lentiviral constructs were made using a human immunodeficiency virus-derived system^50^. Mouse Hsp25 or GFP cDNA were cloned into the transfer vector and cotransfected into HEK293T cells along with the envelope plasmid (pMD2G) and packaging plasmid (pCMV R8.74). Viral stocks were concentrated by sucrose density centrifugation, resuspended and dialyzed against Optimem medium. Packaged virus was titrated on YAMC cells by a cytopathic effect (rounding).

### Immunohistochemical staining

Immunohistochemical staining was performed on sections from formalin-fixed, paraffin-embedded mouse colon for smooth muscle actin, Hsp25, and Wnt-5a or Ki-67 using the Vectastain Elite ABC kit (Vector Labs, Burlingame, CA) or EnVision+System (DakoCytomation, Australia). Slides were counterstained with hematoxylin.

### *In vivo* gene silencing using siRNA injection into mouse colon

To reduce Wnt-5a expression *in vivo*, a modification of *in vivo* silencing was used^50^ (University of Chicago IACUC protocol 71788). All methods were carried out in accordance with relevant guidelines for surgery including anesthesia prior to surgery and health monitoring guidelines which are standard post surgery. The abdominal wall was surgically opened and a section of the proximal colon injected with Wnt-5a silencing or scrambled siRNA. India ink was included so that the margins could be observed. The colonic segment was then returned to the abdominal cavity and closed by surgical stapling. After 4 days, mucosa was harvested from the India ink marked areas and processed for Western blotting and immunohistochemistry.

### *In vivo* Hsp25 lentivirus delivery to colonic mucosa

Mouse experiments were approved by the University of Chicago IACUC Committee protocol 71851. All methods were carried out accordance with relevant guidelines for surgery for anesthesia prior to surgery, introduction of lentivirus to mice, individual housing of mice with lentivirus in biosafety facilities, and health monitoring guidelines post surgery. To induce colonic epithelial cell Hsp25 expression *in vivo*, the mouse abdominal wall was surgically opened and 1 cm section of the cecum clamped. Murine Hsp25- or green fluorescent protein-encoding lentivirus was introduced in into the mouse colon lumen in PBS and left for one hour. The segment was unclamped, returned to the abdominal cavity, and the abdominal wall closed. After 3 days, a section at the site of lentivirus introduction was removed and fixed for immunohistochemical analysis.

### Preparation of cultured colonic epithelial YAMC cells for measurements of β-catenin nuclear localization

YAMC cells were treated with silencing RNA oligonucleotide to mouse Hsp25 using the Silentfect (BioRad, Hercules, CA). When appropriate, cells were infected with Hsp25 encoding lentivirus for 24 hours. Treatment with VUPF CM or Wnt-5a synthesized peptide was initiated immediately upon switching to 37 °C. When appropriate, either synthesized peptide was incubated with anti-Wnt-5a or goat IgG for 24 hours before addition to cells. Cells were harvested using the NE-PER kit (ThermoPierce, Rockford, IL). Protein was measured in the nuclear and cytoplasmic fractions and analyzed for β-catenin by Western blots.

### TOPFLASH luciferase reporter to assess activation of β-catenin

YAMC cells were transfected with TOP or FOP FLASH reporters using LT-1 (Mirus, Madison, WI). Conditions used were as described for β-catenin translocation. Cells were harvested and luciferase activity measured according to manufacturer’s instructions (Promega, Madison, WI).

### Dextran sodium sulfate induced colitis

Mouse treatments were approved by the Kyoto Prefectural University Animal Care and Use Committee protocol M25-150. C57Bl6 mice were purchased from Shimizu Experimental Animals (Osaka, Japan). Mice were provided 2.5% (wt/vol) DSS in drinking water from days 0–7 and regular water days 8–15. On days 8–14, mice were injected i.p. daily with 10 μg Wnt-5a peptide or saline vehicle. Body weights were measured on days 0, 4, 7, 1, 13, and 15 and mice sacrificed and colon length measured and harvested for RNA and protein.

### Statistical Analysis

All experiments were repeated at least three times using cells from different passages. Statistical analysis was performed using ANOVA for multivariate analyses and Bonferroni corrections. Data were expressed as means ± SE.

## Additional Information

**How to cite this article**: Uchiyama, K. *et al.* Butyrate and bioactive proteolytic form of Wnt-5a regulate colonic epithelial proliferation and spatial development. *Sci. Rep.* **6**, 32094; doi: 10.1038/srep32094 (2016).

## Supplementary Material

Supplementary Information
