# Supplementary material for: Butyrate and bioactive proteolytic form of Wnt-5a regulate colonic epithelial proliferation and spatial development
Source: Sci Rep. 2016 Aug 26;6:32094. doi: 10.1038/srep32094 (PMC4999796; doi:10.1038/srep32094)
Supplement: Supplementary Information [file srep32094-s1.doc]

Butyrate and bioactive proteolytic form of Wnt-5a regulate colonic epithelial proliferation and spatial development

Kazuhiko Uchiyama, Toshio Sakiyama, Takumu Hasebe, Mark W. Musch, Hiroyuki Miyoshi, Yasushi Nakagawa, Tong-Chuan He, Lev Lichtenstein, Yuji Naito, Yoshito Itoh, Toshikazu Yoshikawa, Bana Jabri, Thaddeus Stappenbeck, and Eugene B. Chang

**Supplemental Figures and Legends**

**Supplemental Figure S1**


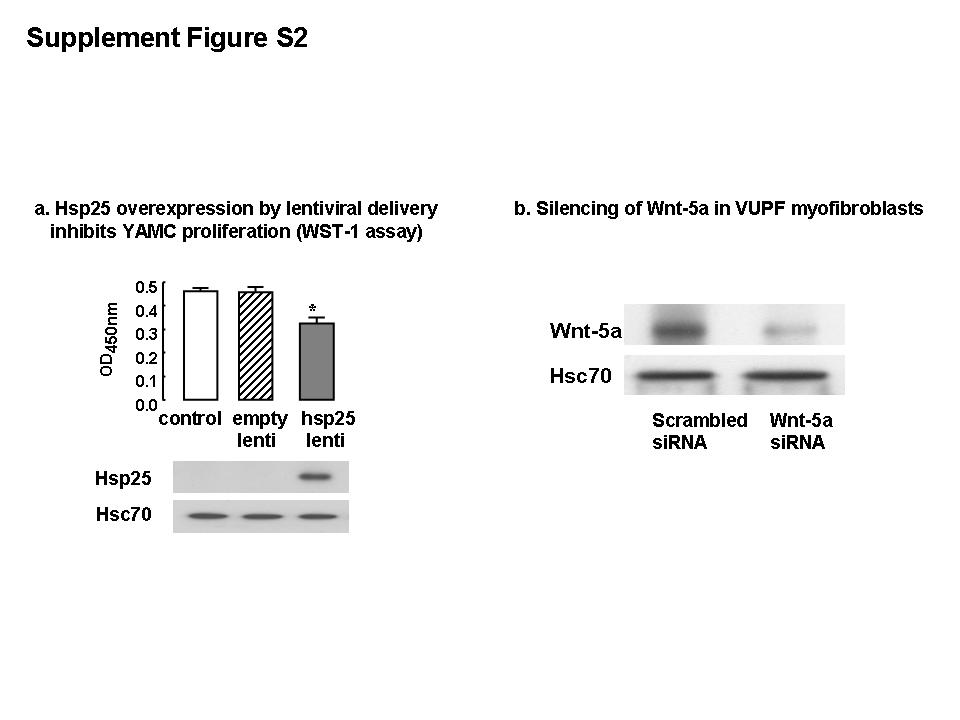


**Supplement Figure S1.** (a) **Increased expression of Hsp25 in intestinal epithelial YAMC cells inhibits their cell growth.** YAMC cells were infected with a lentivirus with a Hsp25 transgene or lentivirus-GFP. Cells were harvested for Western blot analysis of Hsp25 expression (lower panel) or labeled with WST proliferation reagent and cell numbers measured (upper panel). For WST proliferation assay, control indicates no viral infection. Results are representative of three separate experiments. * p < 0.05 (p=4) compared with either control or mock by analysis of variance using a Bonferroni correction. (b) **Validation of RNA silencing of VUPF Wnt-5a production**. VUPF myofibroblasts were treated with either an oligonucleotide to silence Wnt-5a or a scrambled (same composition, different order) oligonucleotide. The immunoblot indicates effective silencing of Wnt-5a. Cells were harvested and analyzed for Wnt-5a and heat shock cognate Hsc70 as a loading control. Image is representative of three separate experiments.

**Supplemental Figure S2**

**
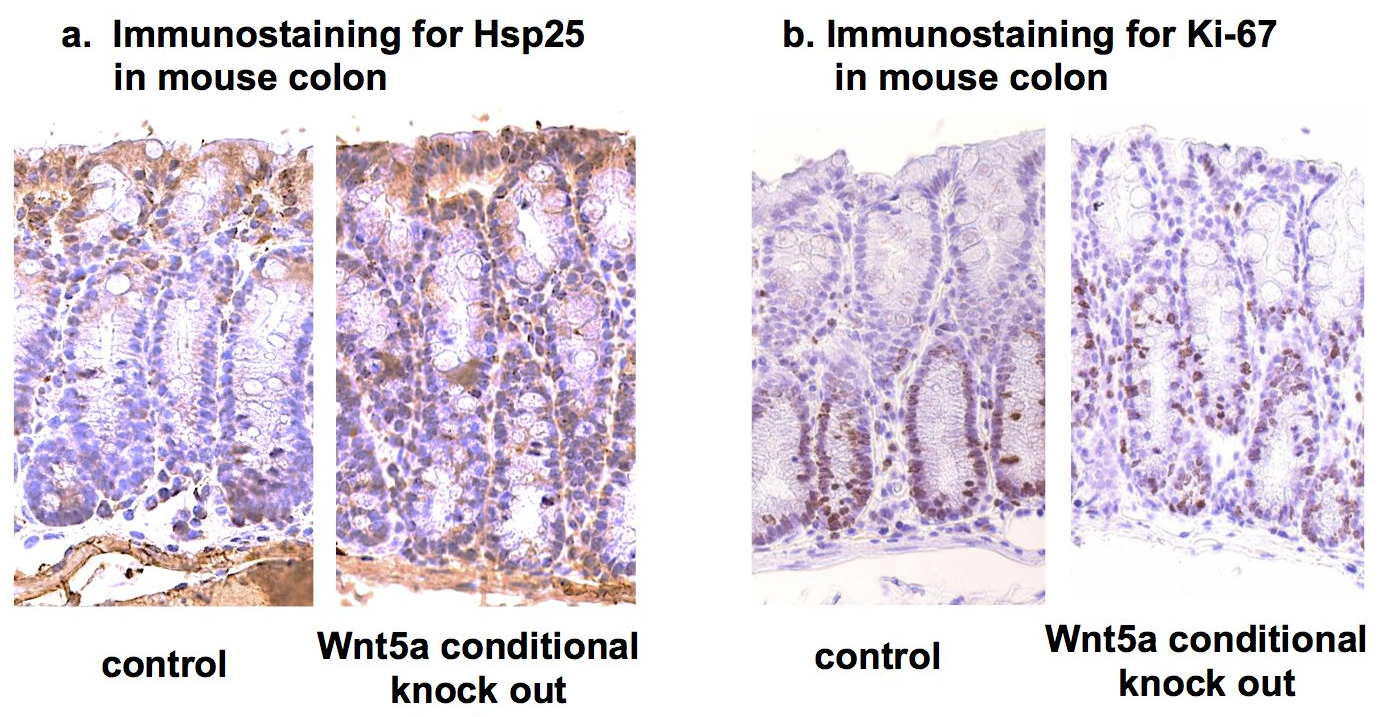
**

**Supplemental Figure S2. Effect of Wnt-5a knockout out on Hsp25 and Ki-67 immunostaining.** Colonic tissues were fixed in formalin and then stained with polyclonal anti-Hsp25 (a) and Ki-67 (b) antibody for control and Wnt5a conditional knockout mice.

**Supplemental Figure S3**


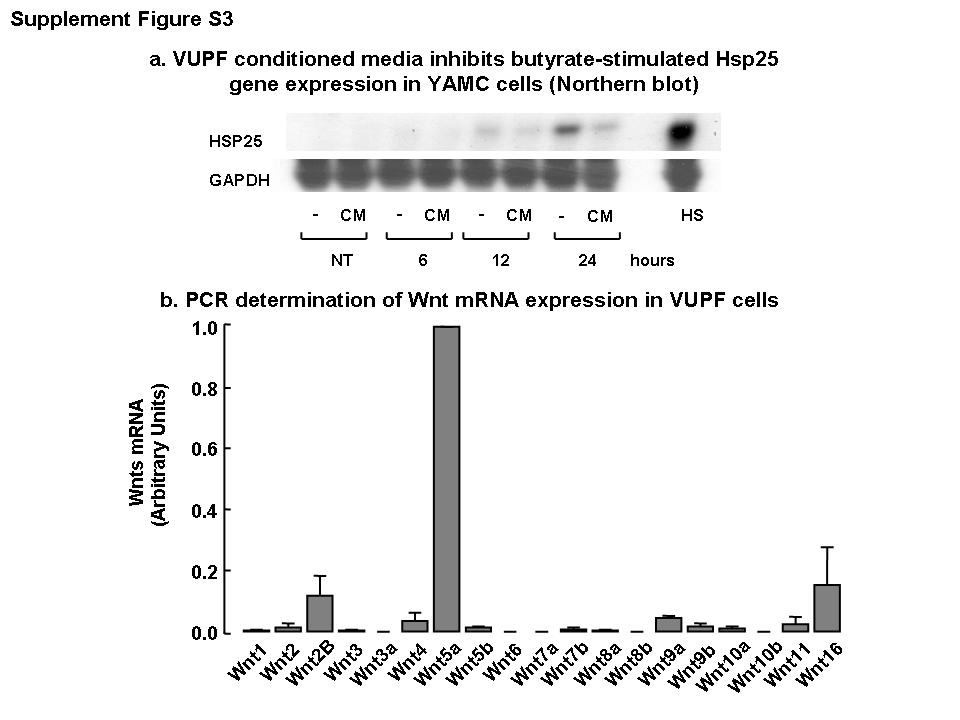


**Supplement Figure S3.** (a) Representative Northern blot showing a time-dependent increased in Hsp25 mRNA expression in YAMC cells (Y) treated with butyrate (5 mM). In the presence of conditioned media from VUPF pericrypt myofibroblasts (CM), butyrate-induced Hsp25 mRNA expression is inhibited. HS – Samples from heat-shocked YAMC cells (41.5^o^C x 23 m). (b) VUPF myofibroblasts express a number of Wnt mRNAs. RNA was harvested from VUPF myofibroblasts and analyzed by RT-PCR for a number of Wnt mRNA by RT-PCR followed by quantitative PCR as described in Methods. Data are means ± SEM for three separate experiments. Arbitrary units were shown relative to Wnt-5a expression which was set at 1.0.

**Supplemental Figure S4**


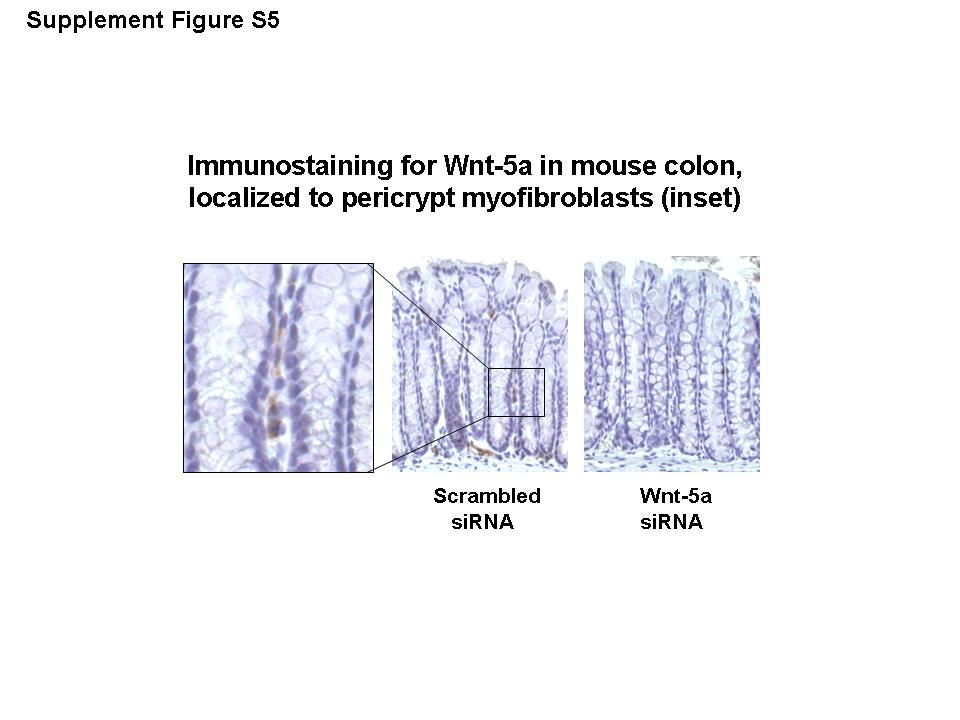


**Supplement Figure S4.** **Wnt-5a mRNA is predominantly expressed in pericrypt colonic myofibroblasts**. Colonic tissues were fixed in formalin and then stained with polyclonal anti-Wnt-5a. The middle panel shows Wnt-5a immunolocalization to pericrypt myofibroblasts (40x), with a magnified view shown in the left panel (200x). Intramural injection of Wnt-5a siRNA of the murine colon results in decreased Wnt-5a protein expression (right panel). Tissues were harvested 72 hrs after intramural injection of Wnt-5a siRNA.

**Supplemental Figure S5**


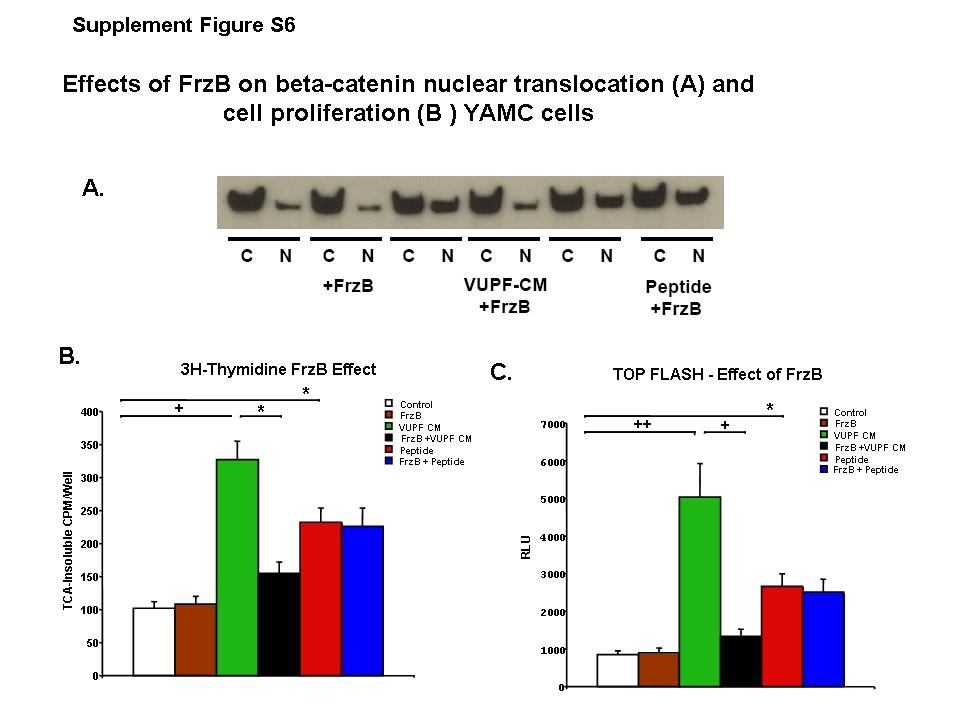


**Supplement Figure S5.** **FrzB does not block Wnt-5a synthesized peptide stimulation of YAMC proliferation or β-catenin activation**. (a) Wnt-binding FrzB does not block β-catenin translocation stimulated by the Wnt-5a synthesized peptide, shown by TOPflash reporter activity in YAMC cells. (b) FrzB also does not block Wnt-5a synthesized peptide stimulation of YAMC proliferation, assessed by 3H-thymidine incorporation. (c) FrzB does not block β-catenin activation (shown by TOPflash) by Wnt-5a synthesized peptide, but does block that stimulated by conditioned media (CM). These different results are likely due to subtle conformation differences between the 36 mer endogenous and 31 mer synthesized Wnt-5a peptides affecting their binding to FrzB.

**Supplemental Figure S6**


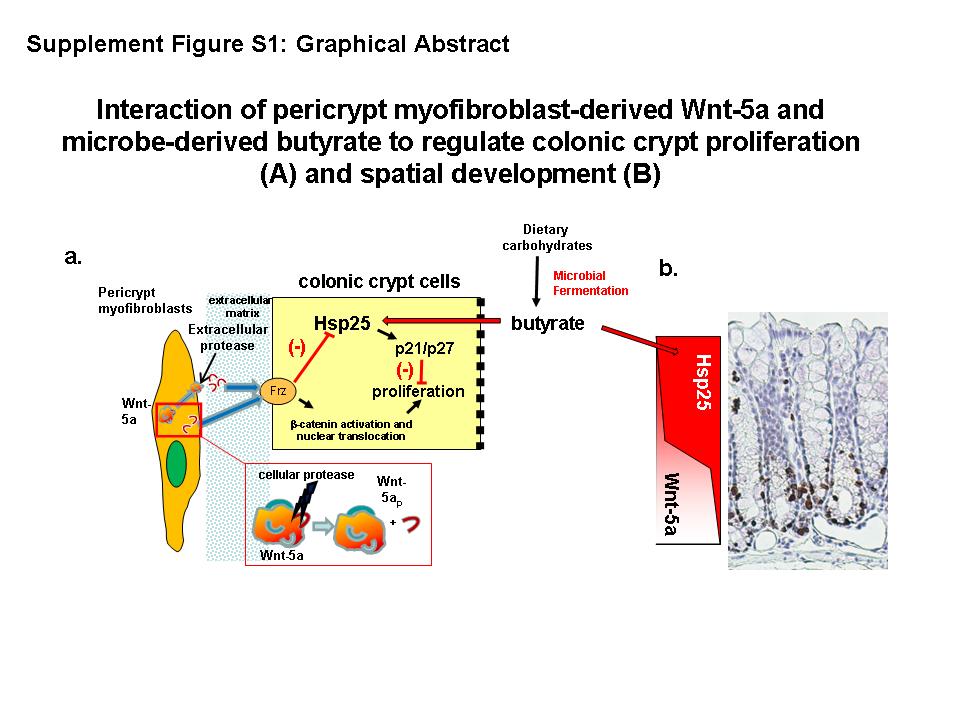


**Supplement Figure S6, Graphical Abstract: Model showing the interactions between stromal pericrypt myofibroblast Wnt-5a and microbe-derived butyrate that regulate colonic epithelial proliferation and spatial development:** (a) In the lower proliferative compartment of the colonic crypt, Wnt-5a is secreted from pericrypt myofibroblasts and is proteolytically modified to a bioactive 36 mer peptide, either intracellularly (inset) or possibly at extracellular sites (e.g. at the cell surface), the latter presumably by tissue tryptases. The more soluble Wnt-5a-derived peptide binds the Frizzled receptor on the epithelial cells (Frz) which activates through a canonical pathway β-catenin, resulting in its translocation to the nucleus. Wnt-5a also down-regulates butyrate-induced cellular proliferation and heat shock protein 25 (Hsp25), the latter serving as an important negative regulator of crypt cell proliferation through increased expression of cell cycle regulators, p21^waf-1^ and p27**.** (b) In the upper half of the colonic crypt where there are far fewer stromal myofibroblast, Wnt-5a levels dramatically diminish, shifting the balance in favor of butyrate-mediated inhibition of cell proliferation and promotion of epithelial cell differentiation.
